# Synergistic Effect of Newcastle Disease Virus and Hydroxyurea in Apoptosis of Human Hepatocellular Carcinoma Cells (HepG2)

**DOI:** 10.1155/bmri/8931051

**Published:** 2026-09-26

**Authors:** Benyamin Baghani, Zahra Sadat Hashemi, Maryam Fazeli, Mahdi Pakjoo, Ehsan Zafari, Hajar Rajaei Litkohi, Ardeshir Ghavamzadeh, Dariush Gholami, Ramin Sarrami Forooshani

**Affiliations:** ^1^ Department of Microbial Biotechnology, Faculty of Biotechnology, Amol University of Special Modern Technologies, Amol, Iran, shomal.ac.ir; ^2^ ATMP Department, Breast Cancer Research Center, National Cancer Institute, ACECR, Tehran, Iran, acecr.ac.ir; ^3^ Department of Nano Biotechnology, Faculty of Biotechnology, Amol University of Special Modern Technologies, Amol, Iran, shomal.ac.ir; ^4^ Cancer and Cell Therapy Research Center, Tehran University of Medical Sciences, Tehran, Iran, tums.ac.ir; ^5^ Department of Bioinformatics and Applied Biotechnology, Faculty of Biotechnology, Amol University of Special Modern Technologies, Amol, Iran, shomal.ac.ir

**Keywords:** combined therapy, hydroxyurea (HU), liver cancer, Newcastle disease virus (NDV), synergistic effect, virotherapy

## Abstract

**Introduction:**

Despite widespread use, chemotherapy often leads to adverse effects and resistance. Hydroxyurea (HU) is commonly used in combination with other chemotherapeutic agents. Efforts have been made to enhance its efficacy and reduce associated toxicity. Combination therapy appears to be the most effective approach for targeting cancer cells through multiple mechanisms. Newcastle disease virus (NDV) has recently been investigated as a potential cancer therapy. This is the first study to evaluate the combined cytotoxicity of NDV and HU in HepG2 cells.

**Materials and Methods:**

The effects of the nonvirulent oncolytic LaSota strain of NDV and HU (alone and in combination) on human liver cancer cells were assessed using MTT, annexin/PI, AO/EtBr staining, scratch and transwell assays, colony formation, quantitative real‐time PCR, and Western blot for gene and protein expression. The combination results were analyzed using the Chou–Talalay method.

**Results:**

The combination of HU and NDV at IC30 doses resulted in approximately 65%–70% cell death, which is significantly greater than that observed with either monotherapy (*p* = 0.0006). The annexin V/PI assay showed that the combination induced 36.8% apoptosis, whereas monotherapy with HU or NDV induced only about 17% (*p* < 0.05). Cell migration was significantly impeded in all treated groups, with the combination resulting in a 65% decrease in migration relative to the control (*p* = 0.009). With HU + NDV, colony formation was reduced by about 60%, and with HU alone, it was reduced by about 43% (*p* = 0.0005). The mRNA expression of VEGF‐A was markedly diminished in all treated groups; but, on the contrary, the cleaved caspase 9 was boosted at the protein level. These data collectively confirm a synergistic inhibitory effect on the proliferation, migration, and survival of HepG2 cells.

**Conclusion:**

The combination of the common chemotherapeutic agent HU with the nonpathogenic LaSota strain of NDV as virotherapy showed a synergistic effect on HepG2 cells in vitro. The results suggest a possible therapeutic benefit by suppressing cancer cell proliferation, inhibiting growth, and inducing cell death. However, these results are restricted to in vitro tests and may not fully represent the complexity of liver cancer in humans; additional in vivo research is required to assess the approach′s safety and clinical applicability. Additionally, this combination might serve as a foundation for further research on multidrug resistance.

## 1. Introduction

Liver cancer is the sixth most common cancer and the fourth leading cause of cancer‐related death worldwide [1]. It can either be primary, originating in the liver, or secondary (metastatic), resulting from the spread of malignancies from other organs. The primary types include hepatocellular carcinoma (HCC) and cholangiocarcinoma, which together represent a major global health burden with limited effective treatment options [2]. Treatment strategies for liver cancer vary depending on the patient′s condition and may include partial hepatectomy, liver transplantation, cryotherapy, thermal ablation, chemotherapy, and radiotherapy [3]. These approaches influence inflammatory mediators, the tumor microenvironment (TME), and cancer immunology [4].

Chemotherapy remains a cornerstone of systemic cancer treatment. It includes not only classic cytotoxic agents but also hormonal and targeted therapies. These agents circulate via the bloodstream and exert effects on cancer cells throughout the body [5]. Systemic therapy is often combined with local treatments, such as surgery, radiotherapy, or hyperthermia, to enhance efficacy [6].

Hydroxyurea (HU), first synthesized in 1967, is a chemotherapeutic drug primarily used for treating hyperproliferative bone marrow neoplasms [7]. HU acts by inhibiting ribonucleotide reductase, leading to cell cycle arrest primarily in the S‐phase, which disrupts DNA synthesis and repair in cancer cells [8]. It has shown efficacy against various solid tumors and is classified as an antineoplastic agent [9]. Moreover, it is associated with relatively mild side effects, and due to its short half‐life, adverse effects typically subside soon after discontinuation. Pharmacokinetic studies indicate rapid urinary excretion, with up to 70% of the administered dose eliminated unchanged [10, 11].

Viral therapy, or virotherapy, has become a new and promising method for treating various human diseases, including cancer. Infection with a virus leads to cytokine changes and immune system stimulation [12–14]. These characteristics encompass the potential for genetic manipulation, the minimal risk posed by certain strains—particularly to healthy cells—and their capacity to selectively infect target tissues [15–17]. In this regard, various viral therapies, including oncolytic viruses (OVs), have been used to target several cancer signaling pathways [18]. OVs include adenovirus, rabies virus, poliovirus, herpes simplex virus, influenza type A virus, measles virus, and Newcastle disease virus (NDV). oncolytic virus therapy (OVT) primarily involves two mechanisms: triggering the host′s cellular immune responses and selectively killing tumor cells [19]. The first report of tumor suppression in humans by OVs concerns cervical cancer, in which apoptosis in cancer cells was observed after inoculation with an attenuated rabies vaccine [20].

NDV is an avian paramyxovirus with a single‐stranded, negative‐sense RNA genome that replicates in the cytoplasm [21]. It is heat‐stable and has antineoplastic properties without damaging human DNA [22]. It has been determined that cancer cells are sensitive to NDV due to a lack of interferon (INF) expression [23]. Researchers who had isolated the 1968 strain of Newcastle virus reported encouraging results in 1999 when they used the attenuated strain in cancer patients. The virus appears to target and replicate specific tumor cell types and does not affect normal cells [24]. The exact mechanism by which NDV suppresses cancer cells is not fully understood. The NDV uses the mitochondrial pathway to cause apoptosis in a variety of cancer cells. Moreover, it leads to a loss of membrane potential in tumor cells, triggering the release of cytochrome C [25]. NDV causes the formation of syncytial cells and disrupts membrane potential, leading to cell necrosis and enhancing the activity of killer immune cells [24, 26]. Some studies have shown that this pathway causes the immune system to initiate an antitumor response [27, 28].

In recent years, the use of the NDV for cancer treatment has been considered; however, challenges remain in achieving optimal therapeutic efficacy with NDV monotherapy. Numerous studies have investigated combining NDV with various chemotherapeutic agents to enhance antitumor efficacy and overcome limitations of monotherapy. For instance, the combination of NDV with doxorubicin has demonstrated synergistic cytotoxic effects in breast and cervical cancer models, attributed to enhanced induction of apoptosis and activation of immune responses [29, 30]. Similarly, NDV combined with 5‐fluorouracil (5‐FU) showed potentiation of anticancer effects by reducing required drug dosages and mitigating systemic toxicity, suggesting improved therapeutic indices [31]. This dual approach can have a more substantial impact, especially in overcoming cancer cell resistance. The main reason why most monotherapies fail to achieve complete remission is the heterogeneity within the tumor cell population. Thus, it can be concluded that the combined use of NDV and various chemotherapeutic agents can target a wider range of cancer cells, with cells resistant to one therapy effectively targeted by the other. These findings highlight the potential of NDV to sensitize tumor cells to chemotherapeutic agents, thereby augmenting their efficacy while possibly reducing adverse effects [32–34].

Indeed, the combined treatment of OVs and chemotherapeutic agents (such as HU) in cancer therapy offers significant benefits. This strategy leverages two distinct yet complementary mechanisms to enhance therapeutic efficacy while reducing cancer cell resistance to treatment. HU can inhibit the defensive mechanisms of cancer cells and increase their sensitivity to viral attacks. Moreover, HU′s ability to arrest the cell cycle at specific stages enhances the virus′s ability to attack and kill cancer cells more effectively [35, 36]. Actually, this disruption in the cell cycle weakens the defensive mechanisms of tumor cells, increasing their susceptibility to OVs. For example, Huang et al. [37] reported that HU treatment increased the cytotoxicity efficiency of adenovirus in human colon and HCC by synchronizing cells at a stage more susceptible to viral infection. They found that HU enhanced viral‐mediated apoptosis in the cancer models, suggesting a synergistic interaction between cell cycle inhibition and viral cytotoxicity.

Another significant advantage of this combination therapy is the robust stimulation of the immune system. OVs can provoke strong antitumor immune responses, and chemotherapeutic agents can amplify these effects. The synergy among the immune system, viral therapy, and chemotherapy leads to more effective treatment outcomes and a reduced likelihood of tumor recurrence. This multipronged approach is particularly important in cancer treatment, as liver cancer and other malignancies often develop resistance to single‐modality therapies. Combining targeted drug therapy with viral treatment offers a novel means to overcome these mechanisms of resistance. The dual action of OVs and drugs like HU—destroying the tumor while simultaneously activating the immune system—provides a promising strategy for enhancing treatment efficacy and minimizing side effects compared with traditional methods. This goal is especially critical given the limitations and resistance associated with current liver cancer therapies. Employing innovative and multidimensional treatment approaches holds promise for improving clinical outcomes in patients. Combining HU with other therapeutic modalities, including OVs, presents an intriguing strategy to enhance anticancer effects and overcome drug resistance. In this study, for the first time, the simultaneous effect of the HU and the NDV on liver cancer was investigated.

## 2. Materials and Methods

### 2.1. Cell Culture

The HepG2 (HCC, Cat No. C158) and Vero (Cat No. C101) cell lines were obtained from the Cell Bank of Iran, Pasteur Institute, and cultured in Gibco′s DMEM supplemented with 10% fetal bovine serum (FBS), 100 *μ*g/mL streptomycin, and 100 *μ*g/mL penicillin. The cells were cultured in a 5% CO_2_ environment at 37°C. Cells from Passages 3 to 5 were used in the experiments. PCR‐based tests were used to screen all cell cultures for mycoplasma contamination before use. As mentioned in our previous studies, primary human fibroblast cells were isolated, purified, and characterized cells according to the protocol we had set up in the laboratory [38].

### 2.2. LaSota Virus Titration and Characterization

The lentogenic strain of NDV, LaSota, was obtained from the Razi Vaccine & Serum Research Institute (Iran), purified by tangential flow filtration (TFF), and then it was grown in 9‐day‐old embryonated eggs. Seventy‐two hours postinoculation (corresponding to 13‐day‐old embryos), the allantoic fluid was harvested. Centrifugation was used to extract and remove debris from the allantoic fluid (3000 rpm for 30 min at 4°C).

Vero cells were seeded at a density of 2 × 10^5^ cells/well in six‐well plates and allowed to adhere overnight. The cells were infected with serial dilutions of LaSota (10^−1^ to 10^−10^) to determine the cytopathic effects (CPE). After 1 h of adsorption, the inoculum was replaced with fresh DMEM containing 2% FBS. This approach allowed for an initial assessment of viral infectivity and cytotoxicity. Mock‐infected cells (treated only with allantoic fluid) served as negative controls. Images were captured at 48‐h postinfection using a phase‐contrast microscope.

The hemagglutination (HA) assay was performed to quantify HA activity of the LaSota preparation and to standardize viral input across experiments; HAU (hemagglutination units) does not directly quantify infectious titer in mammalian cells. Serial two‐fold dilutions of the virus (1:2 to 1:1024) were prepared in phosphate‐buffered saline (PBS) in a 96‐well V‐bottom microtiter plate. An equal volume of 0.5% chicken red blood cells (RBCs) was added to each well, and the plate was incubated at room temperature for 30–45 min. The HA titer was defined as the reciprocal of the highest dilution showing complete HA, resulting in a diffuse mat of RBCs.

### 2.3. Cell Viability Assay

1 × 10^4^ HepG2 cells were placed in 96‐well plates in a complete medium and allowed to adhere overnight. The cells were then treated with different concentrations of LaSota (HA titer), HU, and also LaSota + HU. In fact, NDV was serially diluted from 1024 to 2 HAU. Besides, HU powder (Sigma) was dissolved in cell culture media as the concentrated stock for further analysis. So, it was similarly diluted, starting at 2 mg/mL and decreasing by 0.2 mg/mL per step until reaching 0.8 mg/mL. 3‐(4, 5‐dimethylthiazol‐2‐yl) 2, 5‐diphenyl tetrazolium bromide (MTT) assay for the quantification of cell viability was done. For each NDV concentration, a corresponding control well containing the same volume of allantoic fluid was used, and the test result for that concentration was normalized to that control well. After 24, 48, and 72 h of incubation, staining was performed according to the standard protocol. Finally, the absorbance at 490 nm (OD490) was measured using a microplate reader. All experiments were performed in triplicate. Untreated cells served as the negative control (0% mortality), and cells treated with DMSO served as the positive control (100% mortality).

### 2.4. Annexin V–FITC and PI Binding Assay

A total of5 × 10^5^cells in the six‐well plate were seeded in complete medium and allowed to adhere overnight. Afterward, the cells were treated with HU, LaSota, and LaSota + HU in triplicate. After 24 h of incubation, cells were detached with 0.25% trypsin and then resuspended in 200‐*μ*L binding buffer (Roche apoptosis kit). The cells were incubated with 5‐*μ*L annexin V–FITC for 30 min at room temperature in the dark, followed by 5 *μ*L PI for 5–15 min. Flow cytometric acquisition was performed on an Attune NxT cytometer, and 100,000 events were analyzed per sample, followed by unstained, single‐stained, and compensation controls. The gating strategy included: (i) exclusion of debris using FSC/SSC plots, (ii) doublet discrimination, and (iii) separation of populations into viable (Q4: annexin V^−^/PI^−^), early apoptotic (Q3: annexin V^+^/PI^−^), late apoptotic (Q2: annexin V^+^/PI^+^), and necrotic (Q1: annexin V^−^/PI^+^) cells. Data were processed using FlowJo 10.8 software.

### 2.5. AO/EB Double Staining Assays

Acridine orange (AO) and ethidium bromide (EtBr) dye mixture (AO: 100 mg/mL; EtBr: 100 mg/mL in PBS) was mixed with treated and untreated cells and then visualized by fluorescence microscopy (Nikon Eclipse Ti; ×400 magnification, exposure time: 200 ms). Cells were categorized based on fluorescence characteristics as follows: (i) living cells, which were green in color and carried a normal nuclear structure; (ii) early apoptotic cells, which were yellow–green and had a condensed or broken nucleus; (iii) late apoptotic cells, which were orange in color and had a condensed or broken nucleus; and (iv) necrotic cells, which were orange–red color and had a normal nuclear structure [39]. AO/EB staining was used as a qualitative method.

### 2.6. Scratch Assay

HepG2 cells (5 × 10^5^ cells/well) were plated in DMEM media containing 10% FBS on six‐well plates. After overnight incubation for growing to 80% confluence, the monolayers were scratched with a needle. The cells were washed twice with PBS, and images were taken immediately after scratch creation (0 h) to document the initial wound width [40]. PBS was removed, and cells were treated with HU, LaSota, and HU + LaSota in an optimized growth medium with 10% FBS. After 24 h incubation, the medium was removed and replaced with 1 mL PBS to capture postmigration images. Images were acquired using a Microscope (Leica DM750, Germany) at 40x magnification. Quantitative analysis of wound closure was performed using ImageJ (NIH, United States) by measuring the wound area at 0 h (as 100%) and 24 h. The percentage of wound closure was calculated based on the change in wound area between the two time points. Every experiment was run at least three times.

### 2.7. Migration Assessment

The principle of this test is based on two chambers of a Transwell (8 *μ*m, Millipore, Billerica, Massachusetts, United States) separated by a porous membrane, through which the cells are transferred. In general, the cells were starved for 24 h. Then, the cell suspension (2 × 10^4^ cells) was seeded at the top of the chamber in serum‐free media. This volume of the medium was adjusted up to 200 *μ*L, which contained HU (IC30), or LaSota (IC30), or HU (IC30) + LaSota (IC30) in different groups. Six hundred microliters of serum‐containing medium was introduced as a chemoattractant into the lower chambers. After 24‐h incubation, the protocol was adapted from a previously published study [41]. In brief, the supernatant of the chamber was drained, and the chamber was washed twice with PBS. A cotton swab was used to collect the nonmigrated cells that remained on the upper portion of the filter after the cells that had gone through the membrane were fixed (4% paraformaldehyde). Then, the migrated cells were stained with 2% crystal violet solution for 2 min. After washing, the number of migrated cells was determined and analyzed using ImageJ software.

### 2.8. Colony Assay

To measure the colony formation, 2 × 10^3^ cells (untreated and treated with HU, LaSota, and HU + LaSota) were seeded in 12‐well plates. A colony was defined as a cluster consisting of 50 cells. They were kept under these conditions for 2 weeks to simulate the long‐term culture conditions. Depending on the growth conditions, the culture medium was replaced every few days. The IC50 values of HU were used to define treatment conditions, but the cotreatment of HepG2 cells was conducted at combined IC30 concentrations to enhance efficacy and reduce cytotoxicity. After the incubation period, based on the previously adapted protocol, the resulting colonies were fixed and stained. Each well was stained with 0.5 mL of 2% crystal violet solution for 15 min at room temperature [42, 43]. 10% acetic acid was used to dissolve the crystal violet dye (37°C shaker for 30 min), and the optical density (OD) was finally measured at 545 nm using an ELISA reader.

### 2.9. Real‐Time PCR

RNA extraction was performed using the Trizol method with a commercial kit (DNAbioTech, Iran). The optical absorbance was measured using a NanoDrop spectrophotometer (DeNovix DS‐11) to assess RNA quality and determine its concentration. cDNA synthesis was also performed using a kit (Parstoos Company, Iran). Real‐time quantitative reverse transcription PCR (qRT‐PCR) for VEGF‐A was performed using an Applied Biosystems StepOnePlus Real‐Time PCR System and SYBR Green Master Mix to evaluate the relative expression levels of the target gene, following the standard protocol [44]. *β*‐actin was used as the internal control gene for normalization. Also, relative gene expression levels were calculated using the Pfaffl method in triplicate. Primer sequences used in this study were as follows: VEGF‐A: forward 5 ^′^‐TGCAGATTATGCGGATCAAACC‐3 ^′^ and reverse 5 ^′^‐GCTTCAGGGTCACACTCCAGAA‐3 ^′^; *β*‐actin (internal control): forward 5 ^′^‐AGAGCTACGAGCTGCCTGAC‐3 ^′^ and reverse 5 ^′^‐AGCACTGTGTTGGCGTACAG‐3 ^′^.

### 2.10. Western Blotting

Cells were harvested and lysed using a 2x RIPA buffer containing 20 mM Tris‐HCl, 2% SDS, 100‐mM dithiothreitol, 20% glycerol, and 0.016% bromophenol blue. The lysates were then denatured, and the proteins were separated on a 10% SDS‐PAGE gel. Subsequently, the resolved proteins were transferred onto a PVDF membrane. The membranes were blocked with skim milk for 1 hour at room temperature. Following this, they were incubated overnight at 4°C with primary antibodies. Afterward, the membranes were treated with secondary antibodies (Abcam) for 2 h at room temperature. Protein bands were detected using the DAB (3,3 ^′^‐Diaminobenzidine) substrate.

### 2.11. Statistical Analysis

Every experiment was run at least three times, with triplicates for each group. Data processing utilized the SPSS software (mean ± SD). For multigroup comparisons, one‐way ANOVA was performed, followed by Tukey′s post hoc test to determine significant differences between groups. Statistical significance was defined as  ^∗^ *p* < 0.05,  ^∗∗^ *p* < 0.01, and  ^∗∗∗^ *p* < 0.001. The Chou‐Talalay method was used to evaluate the interaction between HU and LaSota treatments by calculating the combination index (CI) values using CompuSyn software. A CI value less than 1 indicates synergism, a value equal to 1 indicates an additive effect, and a value greater than 1 indicates antagonism [45].

## 3. Results

### 3.1. Combined Treatment With LaSota and HU Significantly Reduces HepG2 Cell Viability

**Figure 1:**
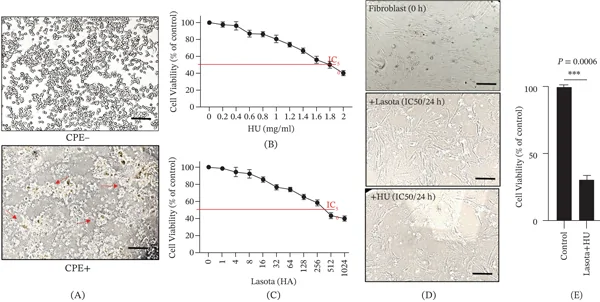
Cytotoxicity of LaSota and hydroxyurea. (A) The cytopathic effects (CPE) consistent with NDV (LaSota) infection (CPE+) in treated Vero cells by NDV. CPE− is control cells without NDV treatment (scale bar: 100 *μ*m). Diagram of cell death in the MTT test, expressed as a percentage, to determine the IC50. The treated HepG2 cells were examined for 24 h, and graphs were drawn to determine the desired doses of (B) hydroxyurea (1.8 mg/mL) and (C) LaSota (350 HA). (D) Treatment of the fibroblast cells by LaSota or HU based on IC50 concentration for HepG2. After 24 h, it indicated no significant toxicity (scale bar: 100 *μ*m). (E) The results of cotreatment based on 1.3 mg/mL HU + 120 HA NDV as IC30, by the MTT assay for HepG2. It indicated significant toxicity as 65% cell death. Data were presented as mean ± SD of three separate experiments, *n* = 3,  ^∗^ *p* < 0.05,  ^∗∗^ *p* < 0.01, and  ^∗∗∗^ *p* < 0.001 versus untreated (control) group.

The LaSota strain of NDV was harvested from embryonated eggs. The CPEs, including cell rounding, syncytia formation, detachment, and cell lysis, were consistent with the well‐documented effects of NDV infection and confirmed that the viral preparation was active and infectious (Figure 1A). NDV HA activity was quantified by HA assay (1024 HAU), which was used to standardize viral input for in vitro experiments. Following the standard protocol of implanting cells and performing the MTT assay, all treatment groups exhibited cytotoxic effects at 24‐, 48‐, and 72‐h posttreatment. However, no significant difference was observed between 24 and 48 h for LaSota. Therefore, 24 h was selected for further analyses. Dose–response curves (diagram of cell viability) for HU and NDV are shown in Figure 1B,C, with half‐maximal inhibitory concentration (IC50) values of 1.8 mg/mL and 350 HAU units, respectively. Treatment of noncancerous (normal) cells with IC50 values of HU and LaSota after 24 h showed preferential cytotoxicity against cancer cells and minimal cytotoxicity against noncancerous cells. LaSota treatment did not show significant cytotoxicity in fibroblasts, but the range of cytotoxicity was slightly higher in treatment with HU. Indeed, under 15% decrease in cell viability was observed when fibroblast cells were treated with HU (Figure 1D).

Cotreatment of HepG2 was performed based on the IC30 concentration (the cytotoxicity power as 30% cell death) of each agent, as 1.3 mg/mL for HU and 120 HAU for NDV. As shown in Figure 1E, cotreatment resulted in approximately 65% cell death. The 95% confidence interval for cell death percentage was 60% and 70%, indicating a significant killing effect compared with individual treatments, which was statistically supported by  ^∗∗∗^ *p* < 0.001.

### 3.2. The Effect of Cotreatment

**Figure 2:**
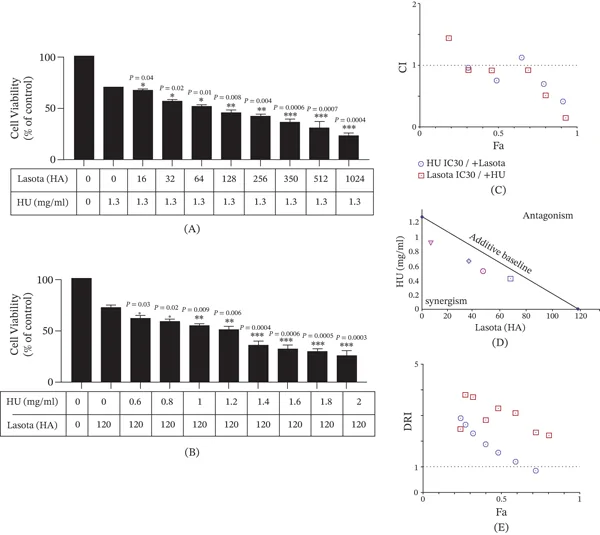
Cotreatment of two effector LaSota and HU. (A) Diagram of HepG2 cell death in MTT test with different concentrations of the LaSota + IC30 concentration of HU as 1.3 mg/mL. Data were presented as mean ± SD of three separate experiments, *n* = 3,  ^∗^ *p* < 0.05,  ^∗∗^ *p* < 0.01, and  ^∗∗∗^ *p* < 0.001 versus untreated (control) group. (B) Diagram of HepG2 cell death in MTT test with different concentrations of the HU + IC30 concentration of LaSota as 120 HA. Data were presented as mean ± SD of three separate experiments, *n* = 3,  ^∗^ *p* < 0.05,  ^∗∗^ *p* < 0.01, and  ^∗∗∗^ *p* < 0.001 versus untreated (control) group. (C) Data analysis (CI), (D) isobologram, and (E) dose reduction index (DRI) charts of the simultaneous effect of dual treatment with the CompuSyn software.

The seeded HepG2 cells in 96‐well plates were administered varying amounts of the LaSota virus, while each well was simultaneously treated with a constant concentration of HU (IC30) (Figure 2A). On the other hand, another plate of seeded cells was treated by different concentrations of the HU (simultaneously treated by a constant concentration of LaSota (IC30) (Figure 2B). The data was given as a query (input) to CompuSyn software for Chou–Talalay method. In this analysis, the CI and fraction affected (Fa) parameters were calculated across a range of concentrations, with values expressed as mean ± SD and 95% confidence intervals from three independent experiments ( ^∗^ *p* < 0.05,  ^∗∗^ *p* < 0.01, and  ^∗∗∗^ *p* < 0.001). The results indicated the CI is less than one, which confirmed the cooperation and synergism effect. The corresponding Fa values represented the fraction of cells affected at each drug combination level (Figure 2C). The isobologram analysis, based on combination concentrations yielding 30% cytotoxicity, is presented in Figure 2D. The synergistic region identified in this graph further confirms the efficacy of the combination therapy. Additionally, the dose reduction index (DRI) was calculated to quantify the extent to which the doses of individual agents could be reduced while maintaining synergistic and therapeutic effects, thereby minimizing potential side effects and optimizing treatment. As shown in Figure 2E, DRI values greater than 1 indicate a successful dose reduction for the respective agents, which is a favorable outcome in combination therapy.

### 3.3. Apoptosis Induction in HepG2 Cells

**Figure 3:**
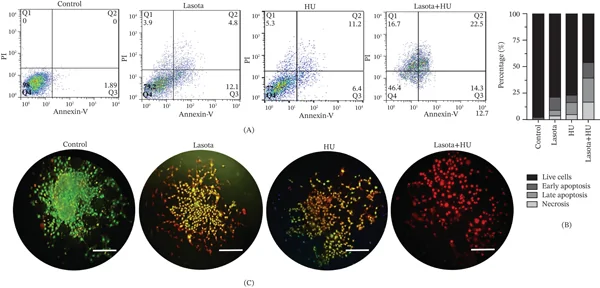
The effect of LaSota on cell apoptosis. (A) The flow cytometry quadrant showed the apoptosis for treated and untreated HepG2 cells. (Q1: necrosis, Q2: late apoptosis, Q3: early apoptosis, and Q4: live cells). The apoptosis percentage (Q2 + Q3) was 16.9%, 17.6%, and 36.8% for HU, LaSota, and LaSota + HU groups, respectively. (B) Apoptosis data were presented as a chart. (C) The apoptotic rate of cells detected by dual acridine orange/ethidium bromide (AO/EB) staining under the fluorescent microscope was not significantly different from that detected using flow cytometry analysis (scale bar: 100 *μ*m). Green, red, and orange fluorescence indicated live, dead, and apoptotic cells, respectively.

The virus and HU induced apoptotic cell death in HepG2 human tumor cells. Following flow cytometry, cell apoptosis was identified using the annexin V‐FITC/PI Apoptosis Detection Kit according to the manufacturer′s instructions. Cells treated with HU alone exhibited approximately 17.5% apoptosis (early and late), whereas LaSota‐treated cells showed 17%. Notably, the combined treatment group demonstrated a significantly higher apoptotic rate of 36.8% (Figure 3AB) (mean ± SD, 95% confidence intervals; one‐way ANOVA with Tukey′s post hoc test).

### 3.4. AO/EB

HepG2 cells were stained with AO/EB. Double staining was observed under a fluorescent microscope. No significant apoptosis was observed in the negative control group (Figure 3C). In the LaSota‐treated group, early‐stage apoptotic cells were detected by crescent‐shaped or granular yellow–green AO nuclear staining. These results were not reported quantitatively. In the HU‐treated group, late‐stage apoptotic cells were also placed with focal and asymmetric staining of EB nuclear orange. Necrotic cells exhibited enlarged morphology with diffuse, intense orange‐red fluorescence throughout the cytoplasm and nucleus, indicating compromised membrane integrity. AO stains all nucleated cells, whereas EB only enters cells with damaged membranes, which makes apoptotic and necrotic cells glow orange–red. Here, the data were examined qualitatively, not quantitatively. Cells were classified as: viable (AO^+^/EB^−^; uniform green nuclei), early apoptotic (AO bright green/yellow with crescent‐shaped or granular chromatin; EB^−^), late apoptotic (AO/EB double‐positive with condensed/fragmented orange nuclei), and necrotic (EB orange–red diffuse nuclear/cytoplasmic staining).

### 3.5. Scratch Assay

**Figure 4:**
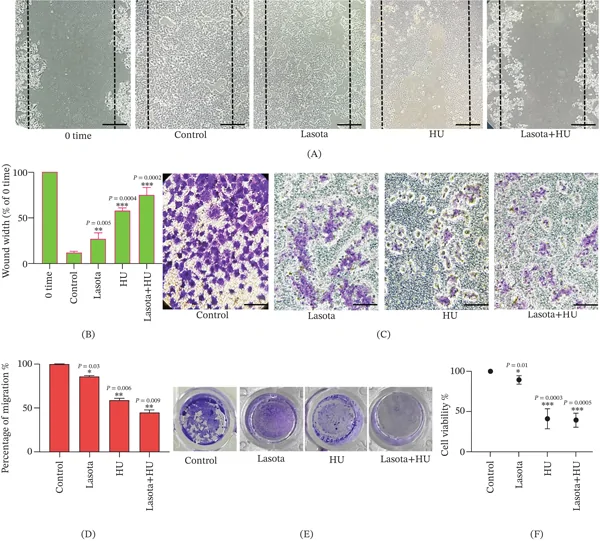
Cell mobility and migration. (A) Wound healing assay: Movement of HepG2 cells derived different groups into the wound was shown for 0 time and after 24 h of scratching (scale bar: 300 *μ*m). (B) Quantification of the wound healing assay based on the wound width at 0 time as 100%. Data were shown from a representative experiment and were presented as mean ± SD of three separate experiments, *n* = 3,  ^∗^ *p* < 0.05,  ^∗∗^ *p* < 0.01, and  ^∗∗∗^ *p* < 0.001 vs untreated (control) groups. (C) Transwell cell migration in treated states and untreated cells (scale bar: 100 *μ*m). (D) The migrated cells were stained with crystal violet and analyzed using ImageJ software. The graph was demonstrated the quantified values of Transwell cell migration assay based on the migrated cells in untreated group as 100%. Data were shown from a representative experiment and were presented as mean ± SD of three separate experiments, *n* = 3,  ^∗^ *p* < 0.05,  ^∗∗^ *p* < 0.01, and  ^∗∗∗^ *p* < 0.001 versus untreated (control) groups. (E) The formation of cell clones in all groups. The reduction of the number of clones in the HU + LaSota group was observed by crystal violet staining. (F) The graph showed the quantified data of colony formation assay (both colony number and size). Data were shown from a representative experiment and were presented as mean ± SD of three separate experiments, *n* = 3,  ^∗^ *p* < 0.05,  ^∗∗^ *p* < 0.01, and  ^∗∗∗^ *p* < 0.001 versus untreated (control) groups.

Figure 4A,B demonstrates that wound healing in the control group was practically complete. But the wound healing in all treated groups was less than in the untreated group. Wound closure was the slowest in cells treated with LaSota + HU compared with the control. Indeed, that vacuity space and wound healing in different groups of virus, drug, and virus + drug were 25%, 58%, and 76%, respectively. This experimental technique reveals the molecular mechanisms that influence cell migration, and the results indicate that cotreatment with LaSota virus as a pharmaceutical agent could suppress and modulate the mobility of cancer cells, thereby advancing treatment.

### 3.6. Transwell Migration Assay

The mobility/migration capacity of treated cancer cells compared with untreated cells has been greatly reduced. Indeed, the untreated cells showed the highest cell migration rate (Figure 4C). For each assay, 2 × 10^4^ HepG2 cells were seeded into the upper chamber of a 24‐well trans well insert and allowed to migrate for 24 h. Each treatment condition was tested in technical triplicate, and the experiment was repeated at least three times. The migration rate was quantified relative to untreated cells as 100% using ImageJ software (Figure 4D). Data are presented as mean ± SD with 95% confidence intervals, and statistical analysis was performed using one‐way ANOVA followed by Tukey’s post hoc test (as  ^∗^ *p* < 0.05,  ^∗∗^ *p* < 0.01, and  ^∗∗∗^ *p* < 0.001).

### 3.7. Colony Formation Assay (CFA)

The CFA test was performed to assess the long‐term effects of drug and virus cotreatment. The data indicated that it has a significant effect in the long‐term. Also, the resulting clones were very small and few in number as a consequence of the LaSota and HU cotreatment (Figure 4E). The CFA was quantified by the amount of crystal violet color produced from stained clones dissolved in 10% acetic acid (Figure 4F). The graph showed more than 60% reduction in colony formation in cells treated with HU + LaSota compared with the control. Data are presented as mean ± SD with 95% confidence intervals from three independent experiments, and statistically significant analysis was performed as  ^∗^ *p* < 0.05,  ^∗∗^ *p* < 0.01, and  ^∗∗∗^ *p* < 0.001 (one‐way ANOVA followed by Tukey′s post hoc test).

### 3.8. Gene Expression Level

**Figure 5:**
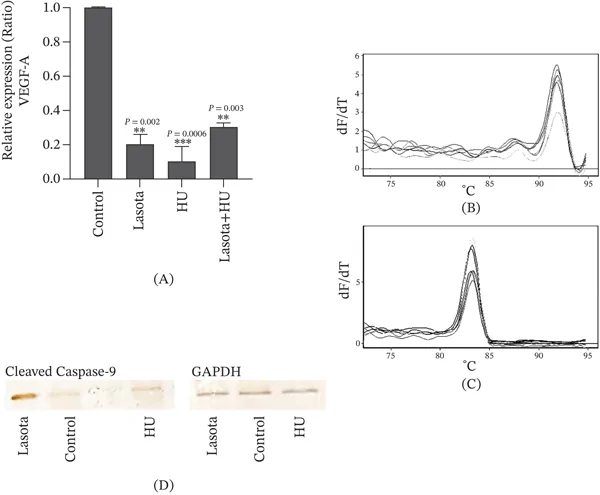
Gene and protein expression levels. (A) The expression levels of VEGF‐A gene were examined in treated groups (LaSota and HU, and cotreatment) compared with the untreated groups by real‐time PCR (Pfaffl ratio) and normalized to *β*‐actin expression level. Data were shown from a representative experiment and were presented as mean ± SD of three separate experiments, *n* = 3,  ^∗^ *p* < 0.05,  ^∗∗^ *p* < 0.01, and  ^∗∗∗^ *p* < 0.001 versus untreated (control) groups. The melting curves of amplicon segments were amplified by (B) *β*‐actin and (C) VEGF‐A primers, respectively. (D) The protein level of cleaved caspase 9 in treated groups compared with the untreated group by Western blot (normalized to GAPDH).

In Figure 5A, the real‐time PCR results indicated downregulation of VEGF‐A gene expression in groups treated with LaSota, HU, and also cotreatment, whereas the LaSota + H–treated group showed no further reduction in VEGF‐A expression (mean ± SD, one‐way ANOVA with Tukey′s post hoc test). The specificity of amplification was confirmed by analysis of melting curves, which demonstrated a single sharp peak for VEGF‐A and *β*‐actin, indicating the absence of nonspecific products or primer‐dimer formation. Suppressing VEGF‐A activity has been shown to decrease proliferation, invasion, and migration while stimulating apoptosis in HCC cells. Figure 5B,C show the melting curves of amplicon segments (PCR products) amplified with *β*‐actin and VEGF‐A primers, respectively.

### 3.9. Protein Expression Analysis

Specific bands corresponding to the protein were detected, confirming the successful expression. The results of the Western blot showed that cleaved caspase 9 protein expression was upregulated in the groups treated with NDV. Caspase 9 is an enzyme that in humans is encoded by the CASP9 gene. It is an initiator caspase, critical to the apoptotic pathway, found in many tissues. The expression of caspase 9, which plays critical roles in apoptosis, could be upregulated or downregulated in all treated groups, but the changes in cleaved caspase 9 levels are more sensitive to apoptosis‐inducing agents compared with cleaved caspase 3, especially when the intrinsic pathway is involved. The higher elevation in protein level of cleaved caspase 9 revealed the ability of NDV to induce the intrinsic apoptotic pathway (Figure 5D).

## 4. Discussion

The primary objective of this research was to evaluate whether virotherapy could complement cancer chemotherapy by enhancing cytotoxic effects through synergistic mechanisms. In recent years, NDV, an avian paramyxovirus, has gained significant attention due to its potent oncolytic activity against various malignancies in mammals. This study investigated the combined effects of NDV (LaSota strain) and HU on human HCC (HepG2) cells using multiple assays, including MTT, apoptosis tests, migration assays, and gene expression analysis. The findings demonstrate that the combination of NDV and HU exerts significant anticancer effects via complementary mechanisms, offering a promising therapeutic strategy for liver cancer.

### 4.1. Cell Cytotoxicity

Our results indicate that NDV and HU induce apoptosis in HepG2 cells through distinct yet complementary pathways. NDV causes tumor cell death by disrupting membrane potential, leading to the release of cytochrome C. A study by Meng et al. [27] has confirmed this result. NDV also primarily activates the intrinsic mitochondrial apoptotic pathway, as evidenced by the release of cytochrome C and activation of caspase 9 and caspase 3, which are involved in cell death [25, 46, 47]. This mechanism aligns with previous studies showing that NDV selectively targets cancer cells due to their impaired INF response [48]. In addition, NDV, by forming syncytial cells and inducing necrosis, significantly mediates antitumor responses [49]. Consistent with previous studies, our results indicate that NDV strain LaSota and HU had no significant effect on normal cultured cells but induced apoptosis of cultured HepG2 cells via a caspase‐dependent pathway [50, 51]. So, these data support the claims of tumor selectivity.

Besides, HU induces cell cycle arrest at the S‐phase by inhibiting ribonucleotide reductase, thereby disrupting DNA synthesis and repair [8]. This disruption renders cancer cells more vulnerable to viral attack, as confirmed by the significantly higher apoptotic rate (36.8%) observed in the cotreatment group compared with monotherapy groups (17% for NDV and HU individually). These findings are consistent with earlier reports demonstrating synergistic effects between NDV and chemotherapeutic agents in breast and colorectal cancer models [18, 20].

The AO/EB double‐staining assay further corroborated these results, revealing early‐stage apoptotic cells (yellow–green fluorescence) in NDV‐treated groups and late‐stage apoptotic cells (orange fluorescence) in HU‐treated groups. Necrotic cells, characterized by diffuse orange‐red fluorescence, were also observed, indicating compromised membrane integrity. These qualitative observations provide additional support for the mechanistic synergy between NDV and HU in inducing cell death.

NDV induces caspase‐dependent apoptotic pathways in HepG2 infected cells. It has been specified in the study that the caspase 9 activity, efflux of cytochrome c, and loss of mitochondrial membrane potential were different at distinct times after NDV infection, but the caspase 3 activity was high both times. So, they concluded that NDV induces apoptosis through the extrinsic apoptotic pathway [52].

Caspase 9 is involved in the caspase activation cascade responsible for apoptosis execution and cleaves/activates caspase 3 and caspase 6. The cleaved caspase 9 was chosen as a key marker in this study. Caspase 9 acts as an initiator caspase in the intrinsic apoptosis pathway, and its activation is directly linked to the formation of the apoptosome complex [53]. NDV, one of the primary agents in this study, is well‐documented to induce apoptosis predominantly through the intrinsic pathway [52]. Therefore, evaluating the activation of cleaved caspase 9 serves as a direct marker to assess the role of NDV in triggering apoptosis. Previous studies have shown that changes in cleaved caspase 9 levels are more sensitive to apoptosis‐inducing agents compared with cleaved caspase 3, especially when the intrinsic pathway is involved [54].

HU functions as a cell cycle inhibitor, causing cell cycle arrest in the S‐phase. This arrest can lead to cellular stress and subsequent activation of the intrinsic apoptosis pathway [8].

The combination of HU and NDV may synergistically enhance the activation of caspase 9, as observed in our Western blot results. These findings indicate that the intrinsic apoptosis pathway is directly affected by this combination. Cleaved caspase 3 acts as an executioner caspase and is activated during the final stages of apoptosis. However, in studies specifically focused on the intrinsic apoptosis pathway, cleaved caspase 9 is often chosen as a primary and more sensitive marker [55]. In this study, our focus was on evaluating the role of the intrinsic apoptosis pathway; hence, cleaved caspase 9 was selected as a key marker. Nevertheless, we explicitly acknowledge in the limitations section that examining cleaved caspase 3 and other apoptosis markers could provide further confirmation of these mechanisms in future studies.

### 4.2. Metastasis and Migration

The qRT‐PCR analysis revealed significant downregulation of VEGF‐A expression in all treated groups, particularly in the cotreatment group. VEGF‐A is a key regulator of angiogenesis, cellular migration, and invasion [56]. Mechanistically, the suppression of VEGF‐A is associated with reduced motility and invasiveness of HepG2 cells, as well as the initiation of apoptosis due to the loss of prosurvival signaling. Although both NDV and HU independently suppressed VEGF‐A expression, the lack of synergy in the cotreatment group suggests that these agents may act through overlapping or independent molecular pathways. For instance, HU primarily inhibits cell proliferation and reduces mTOR activity, whereas NDV suppresses PI3K/Akt and NF‐*κ*B signaling pathways [28, 37]. When one agent already achieves significant suppression of this pathway, the addition of the second agent may not further enhance the effect due to saturation of the target. Furthermore, the distinct mechanisms of action—HU primarily inducing cell cycle arrest and NDV promoting viral‐induced apoptosis—may not converge effectively on the regulation of VEGF‐A, resulting in an additive rather than synergistic outcome. This highlights the context‐dependent nature of drug‐virus interactions, where synergy observed in one biological endpoint (e.g., cell viability) does not necessarily extend to all molecular targets. Therefore, the lack of synergy in VEGF‐A is entirely reasonable and justifiable, suggesting that synergy is a pathway‐dependent phenomenon and is not universal. So, it should not be generalized to all endpoints. This context‐dependent nature of drug‐virus interactions highlights the importance of pathway‐specific synergy, which cannot be generalized across all molecular targets.

These findings are supported by prior studies demonstrating the antiangiogenic and antimetastatic effects of NDV in various cancer models [57]. For example, Kapor et al. [58] reported that NDV suppresses tumor cell proliferation and metastasis by modulating cellular stress responses. Similarly, our scratch and transwell migration assays showed that cotreatment with NDV and HU significantly reduced cell migration by 65%, confirming the antimetastatic potential of this combination. This substantial reduction in cell migration, corroborating the findings of Jabir et al. [47], which indicate that NDV also inhibits migration in breast cancer cells, highlights the potential of NDV as a therapeutic agent in cancer treatment. Likewise, the scratch test was consistent with the results of the Meng et al. [59] study.

### 4.3. Synergy and Long‐Term Effectiveness

The Chou–Talalay analysis revealed a CI of 0.62, indicating a synergistic interaction between NDV and HU. This synergy arises from the complementary mechanisms of action: HU sensitizes cancer cells to viral attack by arresting the cell cycle, whereas NDV promotes viral‐induced apoptosis and enhances antitumor immune responses. The dual targeting of cancer cells through these mechanisms addresses a major limitation of monotherapy, which often fails to achieve complete remission due to tumor heterogeneity. By combining NDV and HU, this approach effectively targets a broader range of cancer cells, including those resistant to individual treatments. Furthermore, the noteworthy aspect is the long‐term effect of this synergy, which could also lead to promising clinical outcomes. In this regard, the CFA demonstrated a 60% reduction in colony formation in the cotreatment group, underscoring the long‐term efficacy of this combination. These results are consistent with previous studies reporting that NDV inhibits colony formation and induces necrosis in hepatoma therapy [60] and colorectal cancer [49].

### 4.4. Previous Studies

This study represents the first investigation of NDV and HU cotreatment in liver cancer. Previous studies have explored similar combinations in other cancer types, such as NDV with doxorubicin in breast and cervical cancer [29, 30] and NDV with 5‐FU in colorectal cancer [31]. Our findings extend these observations by demonstrating significant synergistic effects in reducing cell survival (nearly 70%) and inhibiting metastasis in HepG2 cells. The novel mechanism by which HU arrests the cell cycle and enhances viral infectivity underscores the potential of this combination as an adjuvant therapy for liver cancer.

### 4.5. Limitations and Future Directions

Despite the promising results, this study has several limitations. First, all experiments were conducted in vitro using a single cell line (HepG2), which may not fully represent the complexity of liver cancer in humans. Future studies should validate these findings in multiple cancer cell lines and in vivo models to assess safety, efficacy, and translational relevance. Second, the use of HAU instead of TCID_50_ for quantifying NDV infectious titer may limit precise comparisons across studies. We acknowledge this limitation and plan to incorporate TCID_50_‐based titration in future work to improve dose‐response accuracy.

Another important limitation of the present study is that the kinetics of NDV replication under HU treatment were not directly assessed. Therefore, although the combination showed clear synergistic antitumor activity in HepG2 cells, the extent to which this effect modulates viral replication cannot be determined from the current data. Future studies will include direct measurements of viral replication dynamics, including quantitative analysis of viral RNA and titration of infectious virus, to determine whether HU affects NDV replication or whether the observed synergy primarily reflects complementary cytotoxic mechanisms. Besides targeted delivery systems and applications in other common cancers should also be conducted to broaden the therapeutic scope of this combination.

## 5. Conclusion

The combination of NDV and HU demonstrates significant synergistic effects in suppressing cancer cell proliferation, inhibiting growth, and inducing apoptosis in HepG2 cells. This innovative approach leverages complementary mechanisms to overcome resistance and enhance therapeutic efficacy. Although further in vivo and clinical studies are needed to confirm these findings, this study provides a strong foundation for future research on multidrug‐resistant cancers. The dual targeting of cancer cells through virotherapy and chemotherapy represents a promising strategy to improve patient outcomes and advance liver cancer treatment.

## Author Contributions

**Benyamin Baghani:** conceptualization, formal analysis, methodology, writing – original draft, investigation, writing – review & editing. **Zahra Sadat Hashemi:** conceptualization, formal analysis, methodology, writing – original draft, investigation, writing – review & editing, supervision. **Maryam Fazeli:** conceptualization, formal analysis, methodology, writing – original draft, investigation, writing – review & editing. **Mahdi Pakjoo:** conceptualization, formal analysis, methodology, writing – original draft, investigation, writing – review & editing. **Ehsan Zafari:** conceptualization, formal analysis, methodology, writing – original draft, investigation, writing – review & editing. **Hajar Rajaei Litkohi:** conceptualization, formal analysis, methodology, writing – original draft, investigation, writing – review & editing, Supervision. **Ardeshir Ghavamzadeh:** conceptualization, formal analysis, methodology, writing – original draft, investigation, writing – review & editing. **Dariush Gholami:** conceptualization, formal analysis, methodology, writing – original draft, investigation, writing – review & editing. **Ramin Sarrami Forooshani:** conceptualization, formal analysis, methodology, writing – original draft, investigation, writing – review & editing, supervision.

## Funding

No funding was received for this manuscript.

## Ethics Statement

The study was carried out after obtaining the code of ethics (IR.ACECR.IBCRC.REC.1402.013) and under the direction of the Medical Ethics Committee.

## Conflicts of Interest

The authors declare no conflicts of interest.

## Data Availability

The data that support the findings of this study are available from the corresponding author upon reasonable request.
